# Design of a Machine Learning-Assisted Wearable Accelerometer-Based Automated System for Studying the Effect of Dopaminergic Medicine on Gait Characteristics of Parkinson's Patients

**DOI:** 10.1155/2020/1823268

**Published:** 2020-02-18

**Authors:** Satyabrata Aich, Pyari Mohan Pradhan, Sabyasachi Chakraborty, Hee-Cheol Kim, Hee-Tae Kim, Hae-Gu Lee, Il Hwan Kim, Moon-il Joo, Sim Jong Seong, Jinse Park

**Affiliations:** ^1^Institute of Digital Anti-Aging Healthcare, Inje University, Gimhae, Republic of Korea; ^2^Department of Electronics and Communication Engineering, IIT, Roorkee, India; ^3^Department of Computer Engineering, Inje University, Gimhae, Republic of Korea; ^4^Department of Neurology, Hanyang University Hospital, College of Medicine, Seoul, Republic of Korea; ^5^Department of Industrial Design, Kyoung Sung University, Busan, Republic of Korea; ^6^Department of Oncology, Haeundae Paik Hospital, Inje University, Busan, Republic of Korea; ^7^Department of Neurology, Haeundae Paik Hospital, Inje University, Busan, Republic of Korea

## Abstract

In the last few years, the importance of measuring gait characteristics has increased tenfold due to their direct relationship with various neurological diseases. As patients suffering from Parkinson's disease (PD) are more prone to a movement disorder, the quantification of gait characteristics helps in personalizing the treatment. The wearable sensors make the measurement process more convenient as well as feasible in a practical environment. However, the question remains to be answered about the validation of the wearable sensor-based measurement system in a real-world scenario. This paper proposes a study that includes an algorithmic approach based on collected data from the wearable accelerometers for the estimation of the gait characteristics and its validation using the Tinetti mobility test and 3D motion capture system. It also proposes a machine learning-based approach to classify the PD patients from the healthy older group (HOG) based on the estimated gait characteristics. The results show a good correlation between the proposed approach, the Tinetti mobility test, and the 3D motion capture system. It was found that decision tree classifiers outperformed other classifiers with a classification accuracy of 88.46%. The obtained results showed enough evidence about the proposed approach that could be suitable for assessing PD in a home-based free-living real-time environment.

## 1. Introduction

The most important symptom of Parkinson's disease (PD) is the disturbances in gait that directly affects the daily activities as well as the quality of life [1]. The disturbances in gait characteristics in PD patients are categorized into continuous gait and episodic gait disturbances [2]. Typical features of gait in PD are short-step, hypokinetic, slow gait with decreased arm swing, and episodic gait, which includes freezing of gait (FOG) and festinating gait [3]. Despite the clinical importance, most clinicians usually depend on neurological examination or self-questionnaire-based examination for a short period of time. Therefore, it is very difficult to assess the PD patient's gait status outside the clinic and in a real-world environment. Objective quantification of gait is crucial for the measurement of overall condition as well as disease monitoring in PD. Several clinical scales such as Tinetti mobility test (TMT), Timed Up and Go (TUG), and Unified Parkinson's Disease Rating Scales (UPDRS) are widely used to assess the PD and its severity. In the last decade, numerous studies have investigated the usefulness of gait analysis. Quantitative gait analysis includes infrared-based motion capture (three-dimensional (3D) motion capture), pressure-based gait analysis (GAITRite), and treadmill gait analysis [4–6]. Despite their strength of accurate quantification of gait, clinical implication is still controversial due to high cost and large space or laboratory required for system set up.

To overcome the previous limitations, an attempt has been made in this study to quantify the gait characteristics using the algorithmic-based approach with a wearable accelerometer and its validation using a 3D motion capture system as well as TMT. TMT is widely used for predicting the fall risk of elderly people based on the balance and gait test score. TMT test consists of two components such as the Tinetti balance scale and the Tinetti gait scale. The balance scale consists of 9 parameters, and each parameter has subparameters with a score of 0/1 or 0/1/2. The total possible score of the balance section is 16. The gait scale consists of 8 parameters, and each parameter has subparameters with a score of 0/1 or 0/1/2. The total possible score of the gait section is 12. Each patient has to be assessed based on these two scores. The combined score determines the risk of falls in elderly people. According to Tinetti, a total score of ≤18 is treated as high risk, 19–23 is treated as moderate risk, and ≥24 is treated as low risk [7]. Since the Korean version of TMT has already been validated with the PD patients in the laboratory [8], this version has been used in this study.

The contributions of the proposed study are as follows:

1. This study includes enrolment of a large number of participants with PD, higher than that recommended by the movement disorder society [9]. While the recommended minimum number of patients is 30, this study involves 48 PD patients to provide proper validity and reliability of the result. In addition, 40 healthy older patients' group has been included in the study for the classification of PD subjects from healthy older group based on estimated gait characteristics. Due to a large number of subjects, the proposed study could be recommended for a real-life scenario.
2. The proposed study focuses on the PD patients when they are clinically in “on” state, i.e., after taking dopaminergic medicine. “On” state is the state where the effect of the medicine is present, and the improvement in the gait characteristics is closer to the healthy older group.
3. The good accuracy found by using only accelerometer data for estimating spatiotemporal gait characteristics indicates that the gyroscope data could be excluded for these kinds of studies. This will lead to low power consumption in wearable devices and hence a longer battery life for gait monitoring.
4. The validation study provides a low-cost alternative for assessing gait characteristics in the “on” state of PD patients for both indoor and outdoor environments.
5. The proposed study demonstrates that spatiotemporal gait characteristics estimated by using only accelerometer data are highly correlated with those obtained from a 3D motion capture system. Furthermore, a high correlation was also found between results obtained from the proposed approach and those obtained from the clinical TMT test.
6. The proposed study proposed an automatic system that can classify PD patients and HOG with machine learning techniques based on gait characteristics.

The structure of the paper is outlined in the following way: Section 2 describes the past work related to this study.  Section 3 describes the data collection methods as well as the proposed methodology.  Section 4 presents the results and outcomes of the proposed approach.  Section 5 provides the discussion.  Section 6 describes the conclusion.

## 2. Related Work

The gait analysis performed using a conventional way using a qualitative analysis technique is usually performed in the clinics, and it required a complete medical history of the patients to determine the gait characteristics. The conventional method is relatively simple; however, it depends on the expertise of the physicians, and it is relatively difficult to measure the parameters in a quantitative manner with high accuracy that could be useful for clinical applications. To address this aforementioned problem, a new method has been introduced in this paper to quantify the gait characteristics in an objective way by using quantitative measurement techniques [10, 11]. Wearable devices are now used for a wide range of healthcare observations as well as the measurement of gait. The triaxial wearable accelerometer is known to be a useful tool for assessing gait as well as various motor symptoms in PD. It is not expensive as well as can be used in a comfortable way by the user [12]. Beck et al. proposed a new approach to quantify the gait smoothness using accelerometer and gyroscope signals. They have implemented this method in PD patients as well as healthy controls, and they found clear differentiation in terms of smoothness between two groups. For validation, they have used the correlation technique by comparing their algorithm spectral arc length measure (SPARC) with traditional gait measures and the UPDRS scale. This is one of the potential use cases for using wearable sensors; however, their method did not use 3D motion capture and TMT gait scale for correlation [13]. Hausdorff et al. mentioned that quantification of gait characteristics was possible using a wearable device. They have collected the accelerometer data during the tandem walking and also validated the method. This method also mentioned the potential of using wearable devices for gait analysis. They have not implemented this method to the PD patients, and at the same time, they have not used any other methods such as 3D motion capture or clinical scale for correlation of their method [14]. Gazit et al. proposed a method for quantifying gait initiations using wearable sensors. They have used only one IMU sensor for evaluating the gait initiations and found good results. They have validated the method with the ground truth and found that the interclass correlation coefficient with one wearable sensor ranges from 0.75 to 0.96. They have tested this method on the data collected from younger and older adults. They have not used the 3D motion capture system for validation of their results and also not implemented for PD patients [15]. Anwary et al. proposed a method to find the best location in the foot to place wearable sensors. They have used accelerometer data and gyroscopic data for determining the gait features. For validation of this method, they have used a quality motion capture system. They have done this analysis for healthy groups and mentioned that wearable sensors have the potential to quantify the gait characteristics with high accuracy [16]. Qiu et al. proposed a method that used body-worn sensors to collect the gait data for the assessment of stroke patients. They have found that the gait analysis has a huge contribution towards the diagnosis and treatment of the stroke patients and mentioned that a wearable sensor-based gait analysis system has the potential for supporting rehabilitation in the clinics and hospitals [17]. Byun et al. have proposed a method that uses the wearable accelerometer to measure the gait characteristics of older people having normal cognition. The gait characteristics are quantified using the signal-processing algorithm. Validation of the measurement method was carried out using the GAITRite system. The two methods show a good level of correlation with a correlation coefficient that ranges from 0.91 to 0.96. They have not used the 3D motion capture system, and this method was not tested for PD patients [18]. Pham et al. have proposed a technique that used an inertial measurement system which consists of a gyroscope and an accelerometer to detect the gait patterns such as toe-off and heel strike in the patients with PD as well as older adults when they were encountered with turning as well as straight walking. An algorithm based on continuous wavelet transform is used to detect the gait patterns, and the validation study was carried out using the optoelectronic system. They have not used any clinical scale for comparing the result. 3D motion capture has not been used in this research [19]. Del Din et al. have used the wearable accelerometer for measuring the gait characteristics of older adults as well as PD patients. Signal processing of the collected accelerometer data provides gait characteristics, and the validation was carried out using the instrumented walkway. Fourteen gait characteristics were compared; it was found that four characteristics show a good amount of correlation, another four gait characteristics show an agreement of moderate level, and the rest six characteristics show an agreement of a poor level. This paper does not have any correlation analysis of gait characteristics with the clinical scale, and they did not use the 3D motion capture system [20]. Aich et al. proposed a method that used a wearable accelerometer that can detect FoG, and the validation study was performed that shows a good level of correlation with a correlation coefficient that ranges from 0.961 to 0.984. The study also proposed a machine learning-based approach to distinguish PD with FoG from PD with no FoG, and an accuracy of 88% has been found using SVM classifier. In this research, the effect of dopaminergic medicine has not been considered, and the correlation analysis has not been performed with clinical scale [21]. Mikos et al. proposed a method for FoG detection using a single sensor node. They have developed a system using machine learning based on the extracted features from the signals. They have found a classification accuracy of 92.9% in average of sensitivity and specificity when exploiting its patient adaptive learning capability. This research has given enough evidence that a single sensor can be used for the detection of FoG and machine learning systems for the classification of FoG [22]. Jeon et al. proposed a study that used the wearable device to detect the severity of tremor in PD. The wearable device used in this study consists of an accelerometer and gyroscope. This study also used machine learning techniques to classify the severity of tremor based on the score of UPDRS. It was found that the decision tree outweighs other classifiers with an accuracy of 85.5%. This research has provided enough evidence that the wearable sensors can be used for the diagnosis of PD, and machine learning techniques can be used to automate the system [23]. Samà et al. proposed a study using wearable accelerometer that can detect freezing of gait at real-time environment using a set of features which are related to the previous approaches mentioned by the previous researchers. These features were trained using machine learning classifiers and used to detect the FoG with an improvement over the previous methods. This research suggested that the wearable sensor has the potential to be used for measuring the gait characteristics, and machine learning techniques could be used for the detection of the PD group [24].

The past works mentioned above provide a strong recommendation about the use of the wearable device in the field of PD as well as the effective use of machine learning techniques for autodetection of gait patterns in PD and HOG. The proposed approach has got a lot of inspiration from the previous pieces of literature cited by different researchers. In this study, an algorithmic-based approach has been developed, and it was validated using clinical test and well-known measuring instruments, and a machine learning-based approach has been proposed to detect the PD from the healthy older group using estimated gait characteristics. This system is developed by keeping in mind that it can be used in the home environment as well as in clinical environments.

## 3. Proposed Methodology

### 3.1. Data Collection

This study was performed clinically in the “on” state, i.e., after taking dopaminergic medicine for the PD group of patients. “On” state is the state where there is an effect of the medicine. In this state, there is an improvement in gait characteristics. The resulting gait characteristics are very similar to those of the healthy older group. The accelerometer data for PD patients have been collected in the “on” state so as to study the difference between two groups, i.e., PD patients and healthy older group when they are in a similar state. This study was performed at Haeundae Paik Hospital located at Busan, South Korea. The approval was taken from the review board of the institute (IRB No. 2017-01-028). Prior approval has been taken from all participants before joining this study. The details about the PD group are shown in Table 1.

The healthy older group comprises normal persons with no signs of PD. No medication has been given to them prior to this study. The healthy group consists of 22 males and 18 females. All the subjects in the healthy group were age-matched. The details regarding the patients belonging to the healthy group are shown in Table 2.

UPDRS and H&Y represent Unified Parkinson's Disease Rating Scale and Hoehn and Yahr scale, respectively. UPDRS is widely used for checking the severity of the disease [25]. H&Y scale is a clinical rating scale, which is used to define different categories of motor functions in the PD [26]. Tinetti gait scale is widely used for predicting the fall risk of elderly people.

**Figure 1:**
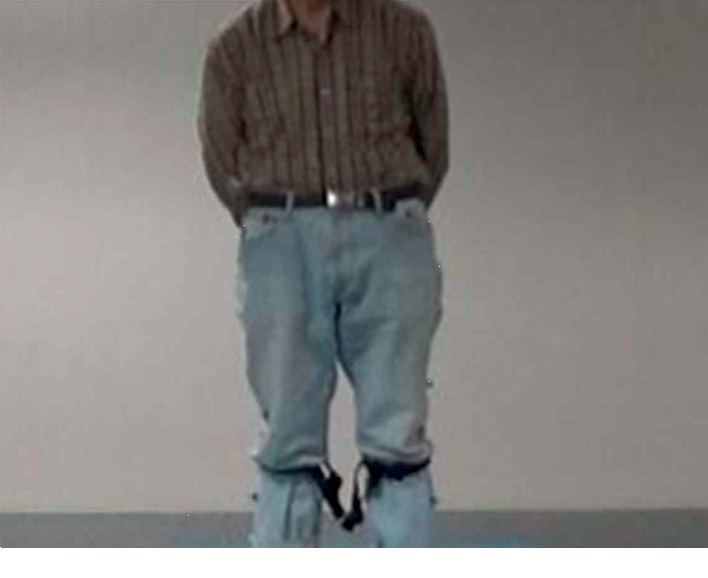
Location of the accelerometers specified for the proposed study.

The participants were asked to wear the accelerometer on the left knee as well as the right knee. Two wearable triaxial accelerometers with a sampling frequency of 32 Hz (Fit Meter, Fit. Life, Suwon, Korea) were used. The triaxial accelerometer measures body movements in all directions: anterior-posterior, mediolateral, and vertical. It is small and lightweight (35 mm × 35 mm × 13 mm and 13.7 gm). It is sensitive to acceleration from −8 g to 8 g, allowing for monitoring of almost all human physical activities. All the participants wore the accelerometers at a distance of 34 cm from the ground, as shown in Figure 1. All the participants were asked to walk along a six-meter track. For validation of the proposed approach, the gait characteristics were also measured by using the 3D motion analysis system (VICON, Oxford, UK). The motion was captured during the walking process. Five important gait characteristics were measured that include step time, stride time, step length, stride length, and walking speed. For estimating gait status more objectively, the Korean version of the Tinetti gait scale [7] was used. The gait characteristics obtained from the 3D motion system and the Tinetti gait scale were used for validation of the proposed approach.

### 3.2. Estimation of Gait Characteristics

**Figure 2:**
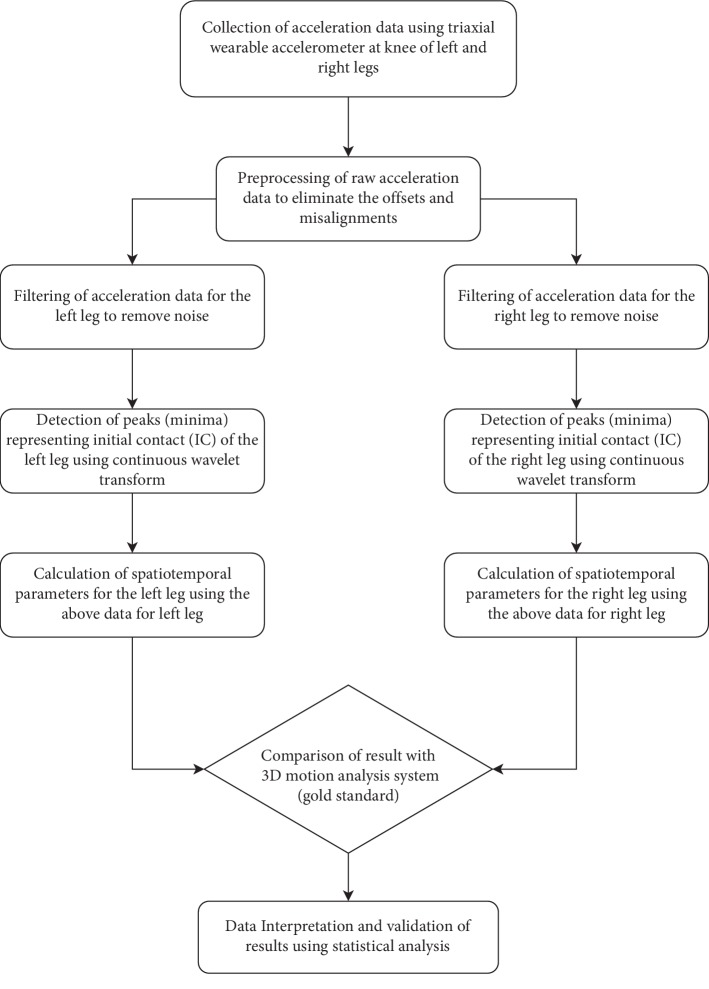
Flowchart of the algorithmic approach for the estimation of gait characteristics.

A variant of the method proposed by Del Din et al. [12] was used to detect the gait cycle. The measured acceleration values along *X-*, *Y-*, and *Z*-axes represent linear accelerations along the medial-lateral (ML), anterior-posterior (AP), and vertical (V) directions, respectively. The corrections are needed to overcome the effect of gravitational component, error due to imprecise position of wearable accelerometer, etc. [27]. The dynamic tilt correction approach proposed in [27] was used to transform the acceleration from ML and AP directions to a global horizontal-vertical coordinate system. The resulting vertical acceleration signal was used hereafter for gait event identification. A low-pass fourth-order Butterworth filter with a cutoff frequency of 15 Hz was used to filter the vertical acceleration signal. The filtered signal was integrated for gait event detection. The objective was to detect the initial contacts (ICs) of the leg, which are also termed as the heel-strike event in a gait cycle. The locations of ICs were detected from the points of minima in the smoothed signal by determining the first-order derivative using the Gaussian continuous wavelet transform. The flowchart of the proposed algorithmic approach based on the accelerometer data is shown in Figure 2.

In this study, five gait characteristics such as step time, stride time, step length, stride length, and walking speed were estimated for the feasibility study of objective assessment of PD using wearable accelerometer data. These five characteristics have received great attention from the researchers in gait-related study and its effectiveness for the assessment of PD. Five major domains of gait study have been proposed by Hollman et al. using the factor analysis: (1) step time and stride time represented by the rhythm domain; (2) temporophasic domain of gait cycle represented by the phase domain; (3) step variability represented by the variability domain; (4) step length, stride length, and gait speed represented by the pace domain; (5) step width represented by the base of the support domain [28]. The aforementioned five characteristics have also been used recently to detect the FoG [20]. Walking speed, stride length, and stride time have been given high importance by Schlachetzki et al. [29] for the discrimination of healthy subjects from the PD subjects. Bertoli et al. estimated the spatiotemporal parameters such as stride time, step time, swing time, stance time, stride length, and gait velocity for the quantitative assessment of PD, mild cognitive impairment patients, and healthy older adults [30].

The step time can be calculated based on the IC events [9] as follows:

(1) $$\begin{array}{c} \text{step time}\left(i\right)=\text{IC}\left(i+1\right)-\text{IC}\left(i\right). \end{array}$$

**Figure 3:**
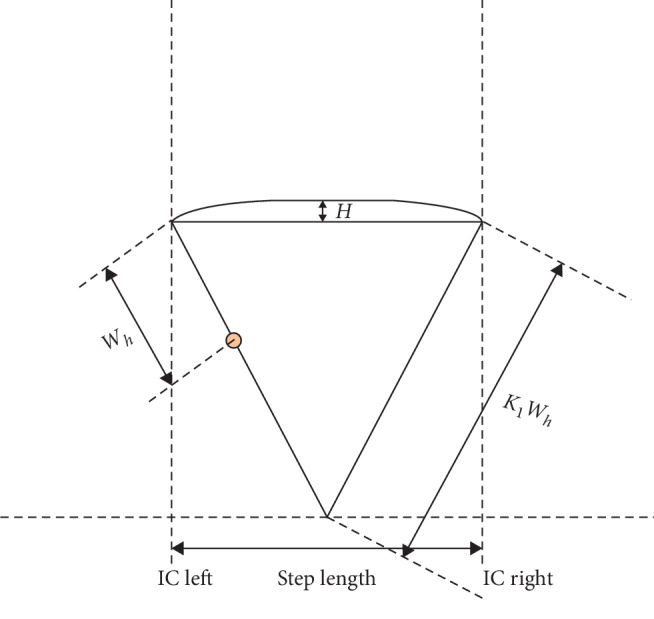
Extended inverted pendulum model [20] for estimation of step length.

Similarly, the stride time can also be computed based on the IC events [9] as follows:

(2) $$\begin{array}{c} \text{stride time}\left(i\right)=\text{IC}\left(i+2\right)-\text{IC}\left(i\right), \end{array}$$

where *i* denotes the index of the IC event in the signal. In the proposed approach, the step length has been estimated using the inverted pendulum model [21, 31], as shown in Figure 3. The step length and stride length can be computed as follows:

(3) $$\begin{array}{c} \text{step length}=K_{I}*2\sqrt{\left(2W_{h}H-H^{2}\right)},\\ \text{stride length}=2*\text{step length,} \end{array}$$

where *W*_*h*_ represents the distance from the ground to the wearable accelerometer and *H* represents the change in height of the wearable sensor between two consecutive IC events. This is computed by finding the difference between the maximum and minimum values of the double integrated vertical acceleration signal between two IC events. The generic multiplying factor *K*_*I*_ is used for mapping the center of mass in an inverted pendulum model with that of the wearable sensor. The value of *K*_*I*_ will change based on the value of *W*_*h*_. Therefore, to avoid the time-consuming task of mapping for each participant that requires determining *K*_*I*_ for each participant, *W*_*h*_ has been fixed at 34 cm, and correspondingly, *K*_*I*_ = 4 has been chosen for this study. Walking speed is calculated as follows [21]:

(4) $$\begin{array}{c} \text{walking speed}=\frac{\text{mean step length}}{\text{mean step time}}. \end{array}$$

These aforementioned five estimated gait characteristics were used as features for the classification of PD groups and healthy older group.

### 3.3. Machine Learning Classifiers and Its Effectiveness for This Study

In this study, comparative performance analysis has been carried out between four machine learning classifiers that have been employed to perform the classification task between the PD patients and the healthy control adults.

#### 3.3.1. The *k*-Nearest Neighbour Classifier (*k*-NN)

The *k*-NN classifier performs the classification process based on the proximity of a data point to the nearest training data points. It generally measures the Euclidean distance to measure the closeness between them. The local data structure has a strong influence on the *k*-NN algorithm. There is no standardized rule to define the value of *k*. The classes are selected based on the majority rule from among the selected number of *k-*nearest neighbors, where *k* is always greater than zero and an integer. The instability in the result, as well as an increase in the variance, can be seen with the smaller values of *k*. The reduction in sensitivity, as well as increasing bias, can be seen with the higher values of *k*. In general, the *k* values are chosen depending on the dataset. In this study, a value of *k* = 5 is chosen as it provides good accuracy [32, 33].

#### 3.3.2. Support Vector Machine (SVM) Classifier

SVM is one of the classifiers suitable to deal with binary classification problems. The classifier tries to maximize the margin arithmetically between two input datasets by defining a surface in an input space, which is multidimensional in nature [34]. In another way, SVM selects the hyperplane with the highest possible margin between two classes while separating them. It is impossible for a hyperplane to separate the data between two classes, but it tries to separate as much data as possible to provide good accuracy [35]. In this study, the radial basis kernel function is used, which provides good accuracy compared to other available kernel functions.

#### 3.3.3. Naïve Bayes (NB) Classifier

NB classifier is one of the simple probabilistic classifiers based on the Bayes' theorem. This classifier selects mutually independent variables. This kind of classifier can be employed in the complex real-life scenario as it can be trained efficiently using the supervised learning technique. The advantage of this algorithm is that it needs less amount of data for training purposes to perform the classification task. In this study, the classification task has been performed by using the Bayes' rule to calculate the probability of class label PD or a healthy group [36].

#### 3.3.4. Decision Tree Classifier

The decision tree classifier works on the basis of conditional statements and its possible consequences. It is a tree-like model. Nodes and branches are the primary components to build a decision tree model. Three steps are followed for building a well-designed decision tree model. The first step is splitting, followed by stopping, and then finally pruning. The continuation of the splitting process stops when the model reaches the desired stopping criteria. The stopping rule is used to avoid the problem of overfitting and underfitting. If the stopping rule does not work well, the pruning method is used to improve the overall classification accuracy [37].

A planned-designed PD detection framework should be efficient and quick enough to perform the binary classification for the classification of PD patients from the healthy older group. Accuracy, sensitivity, and specificity are widely used to measure the effectiveness of the system. The amount of correctness required for the distinction of PD patients from the healthy older group could be measured using the term accuracy. The potential to identify PD is measured by sensitivity, and it is usually expressed as the ratio of true positives to the total number of PD patients [21]. The potential to identify PD when the system identifies the PD can be measured by the term specificity. The subjects belong to the PD group, correctly identified as PD subject, and are represented as true positives. The subjects belong to a healthy older group, correctly identified as healthy older groups, and are represented as true negatives. The subjects belong to the healthy older group but wrongly identified as PD subjects are represented as false positives. The subjects belong to the PD group but wrongly identified as the healthy older group are represented as false negatives. In this study, the objective is to reduce the false negatives as it affects the effectiveness of the system.

## 4. Results

**Figure 4:**
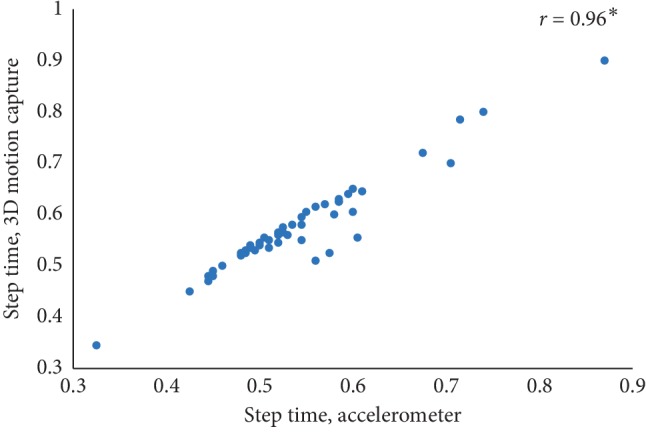
Step time correlation plot between the accelerometer-based approach and 3D motion system (^*∗*^*p* < 0.01).

**Figure 5:**
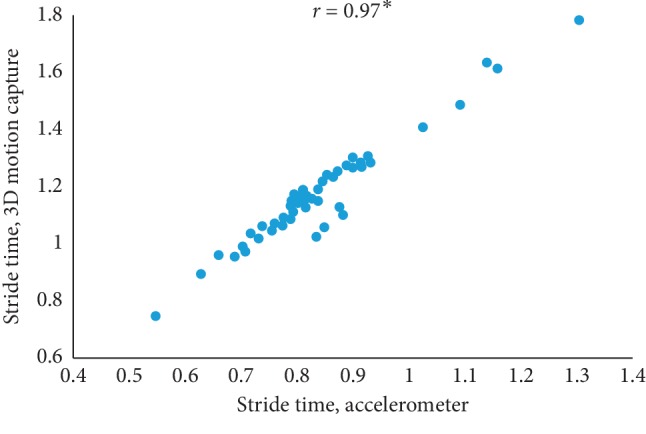
Stride time correlation plot between the accelerometer-based approach and 3D motion system (^*∗*^*p* < 0.01).

**Figure 6:**
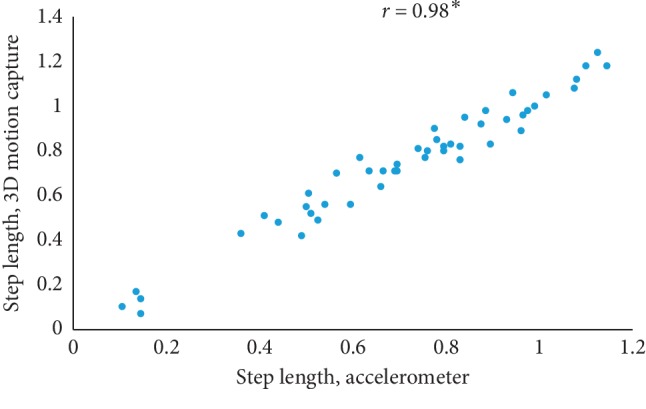
Step length correlation plot between the accelerometer-based approach and 3D motion system (^*∗*^*p* < 0.01).

**Figure 7:**
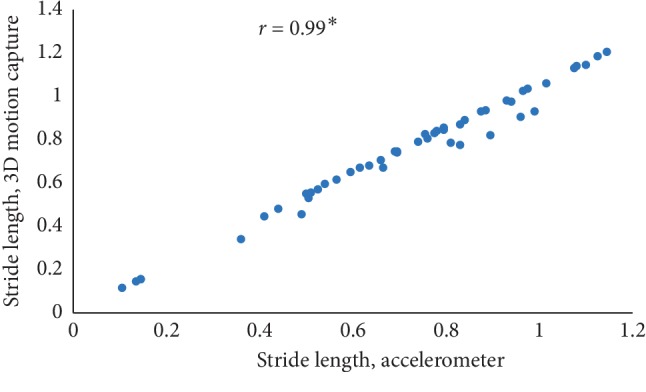
Stride length correlation plot between the accelerometer-based approach and 3D motion system (^*∗*^*p* < 0.01).

**Figure 8:**
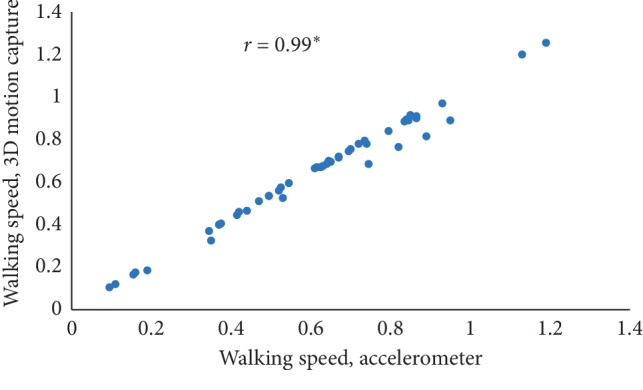
Walking speed correlation plot between the accelerometer-based approach and 3D motion system (^*∗*^*p* < 0.01).

The mean value of five estimated gait characteristics based on the accelerometer data as well as the mean error rate between the algorithmic approach and the 3D motion capture system are highlighted in Table 3. The correlation plots between the algorithmic approach as well as a 3D motion capture system are shown in Figures 4–8. The mean error rate was calculated based on the formula [21] as follows:

(5) $$\begin{array}{c} \text{average error rate}\left(\%\right)=\;\frac{\left(\text{value estimated from acc}\right)-\left(\mathrm{value estimated from}\;3\text{D capture}\right)}{\left(\mathrm{value estimated from}\;3\text{D capture}\right)}*100. \end{array}$$

**Figure 9:**
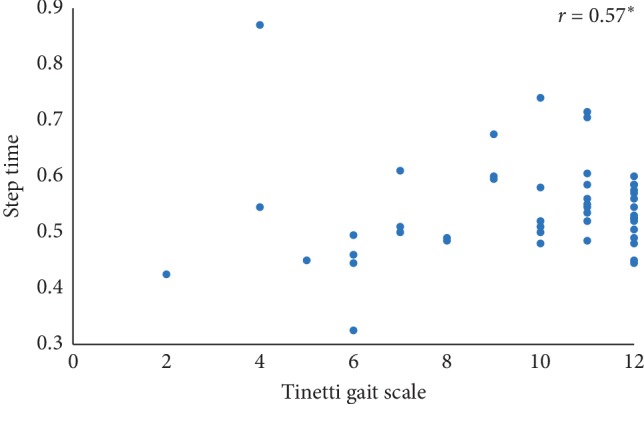
Step time correlation plot between the accelerometer-based approach and Tinetti gait scale (^*∗*^*p* < 0.01).

**Figure 10:**
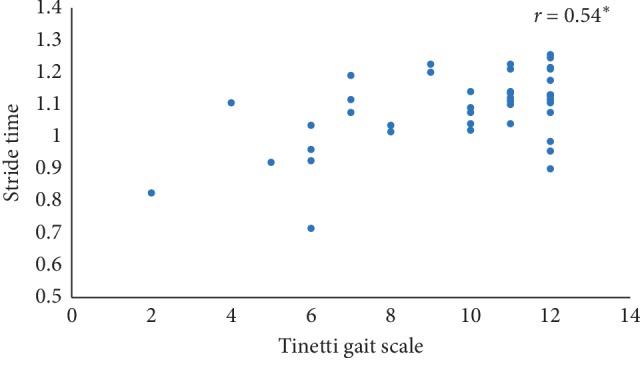
Stride time correlation plot between the accelerometer-based approach and Tinetti gait scale (^*∗*^*p* < 0.01).

**Figure 11:**
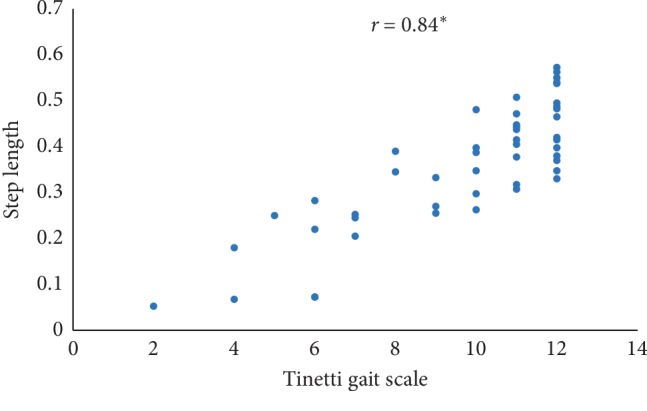
Step length correlation plot between the accelerometer-based approach and Tinetti gait scale (^*∗*^*p* < 0.01).

**Figure 12:**
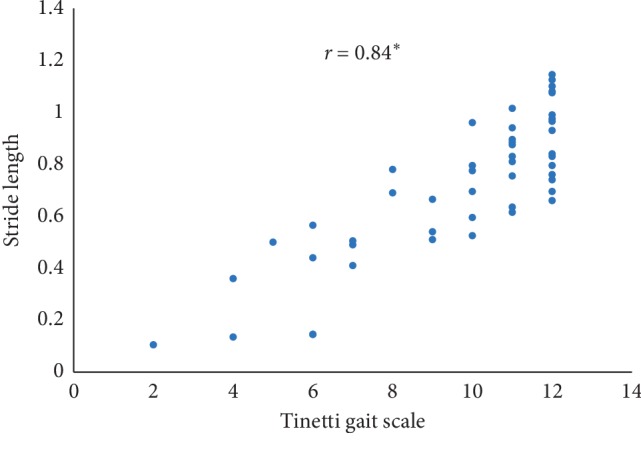
Stride length correlation plot between the accelerometer-based approach and Tinetti gait scale (^*∗*^*p* < 0.01).

**Figure 13:**
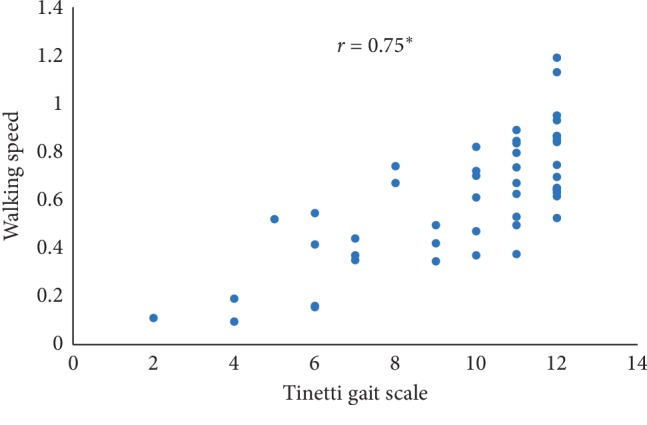
Walking speed correlation plot between the accelerometer-based approach and Tinetti gait scale (^*∗*^*p* < 0.01).

In this paper, Tinetti mobility test (TMT) gait scale is used to assess the spatiotemporal gait characteristics such as step time, stride time, step length, stride length, and walking speed and its importance in terms of clinical practices by comparing the score with the result obtained using other methods, in this case, computerized gait analysis using accelerometer data and 3D motion capture system. The true changes in the gait characteristics can be easily understand based on the accuracy of the clinical observation measures, and it is an important step in clinical practices. So, in this paper, we have used Pearson's correlation coefficient to analyze the relationship between the TMT gait scale score, and spatiotemporal gait characteristics derived objectively used computerized gait analysis using accelerometer data and 3D motion capture system. The correlation plots between TMT gait scale and gait characteristics measured from the 3D motion capture system are shown in Figures 4–8. We have found strong correlations between them, and the results were mentioned as follows: step time (0.96, *p* < 0.01), stride time (0.97, *p* < 0.01), step length (0.98, *p* < 0.01), stride length (0.99, *p* < 0.01), and walking speed (0.99, *p* < 0.01). Similarly, the correlation plots between TMT gait scale and gait characteristics obtained from the wearable accelerometer data are shown in Figures 9–13. We have found moderate to strong correlations between them, and the results were mentioned as follows: step time (0.57, *p* < 0.01), stride time (0.54, *p* < 0.01), step length (0.84, *p* < 0.01), stride length (0.84, *p* < 0.01), and walking speed (0.75, *p* < 0.01).

This study used the split named as stratified train-validation [21] with a ratio of 70 : 30 for training and validation. The total number of subjects including both the groups is 88. Out of 88 subjects, 62 subjects belong to the training group, and the rest 26 belong to the validation group. Out of 26 subjects, which belong to the validation group, 14 subjects belong to the PD group (PDG) and 12 subjects belong to the healthy older group (HOG). Moreover, a 5 split cross-validation was also performed based on the subject's data to check the generalizability of the model. The cross-validation was performed in such a way where the data of 62 random subjects were used to train a classifier and the rest data of 26 subjects were used for checking the testing accuracy. Test set 1, test set 2, test set 3, and test set 4 consist of 26 subjects each. The cross-validation was performed using 4 different classifiers, namely, KNN, SVM, Naive Bayes, and decision tree. The implementation of four different algorithms was done to perform a comparative analysis between the classifiers. After successful cross-validation, it was found that the decision tree plotted the best set of results by prompting a maximum accuracy of 88.46%, sensitivity of 92.86%, and specificity of 90.91%, respectively.  Table 4 shows the results for the cross-validation.

**Figure 14:**
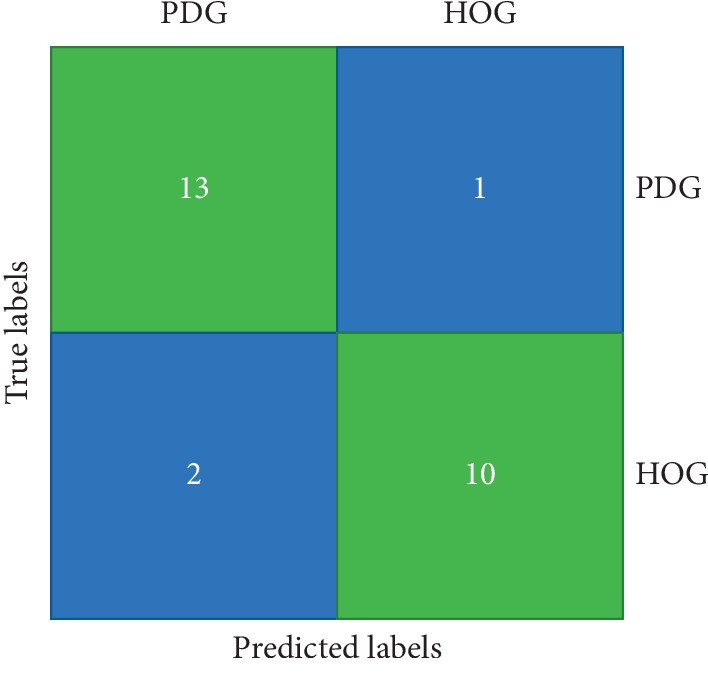
Confusion matrix.

The classifiers' performance has been evaluated using three parameters such as accuracy, sensitivity, and specificity. The classification results are shown in Table 5. The decision tree classifier could able to provide the highest accuracy of 88.46% with a sensitivity of 0.9286 and specificity of 0.9091. From 14 subjects belonging to PDG, the proposed model correctly identified 13 as PDG. Similarly, from the 12 subjects belonging to HOG, the proposed model correctly identified 10 as HOG. The confusion matrix is shown in Figure 14.

## 5. Discussion

This study proposes an algorithmic approach to estimate the gait characteristics of PD subjects as well as the healthy older groups. The approach is validated using measuring instruments and clinical scale. It is also proposed that the machine learning approach can be used for automatic detection and differentiation of PD patients from the healthy older group.

Although wearable sensors have been widely used in many fields, these have not been given enough importance in PD-related assessment due to distorted gait pattern. Sijobert et al. [38] have proposed a technique that provides a mean error rate of 10.3% for the PD group and 6% for the healthy group. The comparison has been made based on the estimated stride length calculated using wearable sensor data and further validated using the GAITRite-based walkway system. The estimated mean error rate for the five gait characteristics is found to be less than 8% with our proposed approach, which used the wearable accelerometer to collect the data. The results of our proposed approach provide the feasibility of our approach when compared with the previous study. The proposed study provides some new ideas that are as follows:

- The results obtained in this study include various phenotypes and severity of PD due to the large sample size.
- This study uses the Tinetti gait scale [7] and the 3D motion capture system [39] for validation of gait status. The previous report [38] has demonstrated the validation using GAITRite that can assess spatiotemporal data by using pressure parameters.
- The gait characteristics estimated using our proposed approach have been compared with the clinical scale, and the result shows a good level of agreement, which makes the method feasible to be implemented in the real-life environment.
- It is observed from the study that only accelerometer data can provide enough information for performing the gait analysis, which leads to redundancy of gyroscopic data, which indirectly saves battery power, time, and cost.

The strength and possibility of the wearable sensor-based PD assessment are aimed at long-term monitoring of gait. Gait disturbance usually gets aggravated in specific cases such as starting time, meeting narrow space, or obstacle [40]. The hospital has limited space, and therefore, it is difficult to replicate gait disturbance observed in a real-world scenario. The battery mounted in our device lasts up to 72 hours. The limitation of this study is that the patients in the relatively early stage of PD were not enrolled, and therefore, this study is not intended at assessing various balance status in PD such as a freeze of gait (FOG) or hypokinetic and short-step gait. The classifiers used in this study have shown good accuracy. All the classifiers showed the acceptable results in terms of performance parameters such as accuracy, sensitivity, and specificity.

## 6. Conclusion

The proposed study highlights the feasibility of wearable accelerometers for gait analysis of PD patients. The algorithmic approach used in this study is able to estimate the gait characteristics with an acceptable mean error rate. The validation study was performed to compare the estimated values from the algorithmic approach with those obtained from the 3D motion capture system and TMT. The proposed approach is a low-cost approach for the detection of PD as well as able to distinguish PD subjects from the healthy older group. It is also observed that the proposed classification model could able to achieve an accuracy of 88.46% with a sensitivity of 0.9286 and a specificity of 0.9091. The objective of reducing the false negatives as much as possible could be achieved. The proposed approach showed enough potential to get recommended for the clinicians to use in the laboratory as well as in the home environment.

In the future, we will collect gait data from a large number of PD patients to summarize the gait characteristics in a better way so that it could be promoted for clinical applications. We would also like to combine brain EEG signals with the gait data to understand more about the relation and detect the symptoms like freezing of gait before it happens. We would like to combine the MRI image with the gait data for more accurate diagnosis and early detection of PD.

## Figures and Tables

**Table 1:** Details of the PD group.

M/F (*n* = 48)	25/23
Age	70.61 ± 9.51
UPDRS part III	20.9 ± 12.31
H&Y stage	2.10 ± 0.74
Disease duration (months)	35.49 ± 27.07
Timed-up and go	20.87 ± 15.78
Tinetti gait scale	9.86 ± 2.56

**Table 2:** Details of the healthy group.

M/F (*n* = 40)	22/18
Age	69.36 ± 7.42
UPDRS part III	0
Disease duration (months)	0

**Table 3:** Mean value of gait characteristics and average error rate for the left and right legs.

Sl. no.	Parameters	Mean value (3D motion capture)	Mean value (algorithm)	Mean error rate (%)
*Left leg*				
1	Step time (s)	0.57	0.54	6.94 ± 2.82
2	Stride time (s)	1.17	1.13	4.76 ± 3.55
3	Step length (m)	0.37	0.34	6.35 ± 2.85
4	Stride length (m)	0.74	0.71	6.51 ± 2.92
5	Walking speed (m/s)	0.64	0.61	7.12 ± 2.74

*Right leg*				
1	Step time (s)	0.54	0.56	7.14 ± 2.52
2	Stride time (s)	1.18	1.14	5.25 ± 3.62
3	Step length (m)	0.37	0.34	6.15 ± 2.81
4	Stride length (m)	0.74	0.70	6.35 ± 2.71
5	Walking speed (m/s)	0.69	0.66	6.72 ± 3.14

**Table 4:** 5 split cross-validation.

Performance (%)	KNN	SVM	NB	Decision tree
Accuracy test set 1	82.11	81.36	84.52	86.28
Sensitivity test set 1	0.8746	0.7801	0.8225	0.9152
Specificity test set 1	0.8452	0.725	0.8654	0.8833
Accuracy test set 2	83.64	84.25	81.20	84.31
Sensitivity test set 2	0.8055	0.8139	0.8558	0.8631
Specificity test set 2	0.8519	0.8687	0.8411	0.8551
Accuracy test set 3	86.32	84.93	85.31	82.28
Sensitivity test set 3	0.9025	0.8755	0.9032	0.8111
Specificity test set 3	0.8947	0.9054	0.8748	0.8364
Accuracy test set 4	85.57	87.23	84.41	88.46
Sensitivity test set 4	0.9125	0.9189	0.8956	0.9286
Specificity test set 4	0.8836	0.8997	0.8735	0.9091
Accuracy test set 5	87.26	84.39	79.32	87.32
Sensitivity test set 5	0.8568	0.8793	0.8178	0.9025
Specificity test set 5	0.9034	0.8998	0.8227	0.9131

**Table 5:** Classification results.

Performance	*k*-NN	SVM	NB	Decision tree
Accuracy (%)	85.57	87.23	84.41	88.46
Sensitivity (%)	0.9125	0.9189	0.8956	0.9286
Specificity (%)	0.8836	0.8997	0.8735	0.9091

## Data Availability

The data used to support the experiments and the finding of the study have been duly included in Section 3 of the paper.  Section 3.1 clearly describes about the data.
